# How Early Can Myocardial Iron Overload Occur in Beta Thalassemia Major?

**DOI:** 10.1371/journal.pone.0085379

**Published:** 2014-01-22

**Authors:** Gaohui Yang, Rongrong Liu, Peng Peng, Liling Long, Xinhua Zhang, Weijia Yang, Shaohong Tan, Hongfei Pan, Xingjiang Long, Taigang He, Lisa Anderson, Yongrong Lai

**Affiliations:** 1 Department of Hematology, the First Affiliated Hospital of Guangxi Medical University, Nanning, China; 2 Department of Radiology, the First Affiliated Hospital of Guangxi Medical University, Nanning, China; 3 Department of Hematology, 303rd Hospital of People's Liberation Army, Nanning, China; 4 Department of Pediatrics, Women and Children Hospital of Guilin, Guilin, China; 5 Department of Pediatrics, Women and Children Hospital of Yulin, Yulin, China; 6 Department of Pediatrics, Affiliated Hospital of Youjiang Medical University for Nationality, Baise, China; 7 Department of Pediatrics, The People's Hospital of Liuzhou, Liuzhou, China; 8 Cardiovascular Sciences Research Centre, St George's University of London, London, United Kingdom; 9 Biomedical Research Unit, Royal Brompton Hospital, London, United Kingdom; Mayo Clinic, United States of America

## Abstract

**Background:**

Myocardial siderosis is the most common cause of death in patients with beta thalassemia major(TM). This study aimed at investigating the occurrence, prevalence and severity of cardiac iron overload in a young Chinese population with beta TM.

**Methods and Results:**

We analyzed T2* cardiac magnetic resonance (CMR), left ventricular ejection fraction (LVEF) and serum ferritin (SF) in 201 beta TM patients. The median age was 9 years old. Patients received an average of 13 units of blood per year. The median SF level was 4536 ng/ml and 165 patients (82.1%) had SF>2500 ng/ml. Myocardial iron overload was detected in 68 patients (33.8%) and severe myocardial iron overload was detected in 26 patients (12.6%). Twenty-two patients ≤10 years old had myocardial iron overload, three of whom were only 6 years old. No myocardial iron overload was detected under the age of 6 years. Median LVEF was 64% (measured by CMR in 175 patients). Five of 6 patients with a LVEF<56% and 8 of 10 patients with cardiac disease had myocardial iron overload.

**Conclusions:**

The TM patients under follow-up at this regional centre in China patients are younger than other reported cohorts, more poorly-chelated, and have a high burden of iron overload. Myocardial siderosis occurred in patients younger than previously reported, and was strongly associated with impaired LVEF and cardiac disease. For such poorly-chelated TM patients, our data shows that the first assessment of cardiac T2* should be performed as early as 6 years old.

## Introduction

Myocardial siderosis remains the most common cause of death in patients with beta thalassemia major (TM) [1]. In affluent countries such as the UK, there has been a dramatic improvement in survival since the introduction of cardiovascular magnetic resonance(CMR) for assessment of myocardial iron [2], but iron induced cardiomyopathy still accounts for the majority of all deaths [3]. Early detection of myocardial iron overload is important because survival is only 50% once overt heart failure is manifest [4] and patients remain asymptomatic until late in the course of development of iron overload cardiomyopathy. Usefully, noninvasive CMR can quantify iron overload in different organs such as the heart and liver even before the development of symptoms [5], [6]. In this regard, CMR relaxometry T2* is being increasingly used worldwide for monitoring transfusion-dependent TM [7], [8].

The guidelines for the clinical management of thalassemia edited by Thalassemia international federation (TIF) recommended that the first assessment of cardiac T2* would be done at puberty for the well-chelated patients who received chelation therapy early and regularly [9], but there is not a specific guideline for poorly-chelated patients. Most TM patients in developing countries including China are poorly-chelated and many of them die in childhood or early adolescence due to the lack of access to specialist care [10], [11]. For poorly-chelated patients, there is limited data on when the myocardial siderosis occurs and when the CMR T2* screening should be initiated. To answer these questions, we designed this study to determine the age when cardiac iron overload occurs and the prevalence of abnormal cardiac MRI T2* and myocardial function on a large young TM population in mainland China.

## Materials and Methods

### Study Population

We studied 201 TM patients (192 children and 9 adults) from November 2010 to January 2013. The median age was 9 (4–25) years old. All patients were transfusion-dependent and required transfusions from early childhood. Thirty-five patients (17.4%) received splenectomy. Five patients (2.5%) were well-chelated and 196 patients (97.5%) were poorly-chelated. Patients were considered to be well-chelated if the following criteria were met [12], [13]: (i) initiate treatment after first 10–20 transfusions or serum ferritin (SF) level above 1000 ng/ml; (ii) chelators dose: Deferoxamine (DFO) is 20–40 mg/kg/day in children and 50–60 mg/kg/day in adults, at least 5 days a week. Deferiprone (DFP) is 75 mg/kg/day. Deferasirox (DFX) is 20–40 mg/kg/day; (iii) good compliance with chelation therapy, defined as a≥90% adherence to the instructions given by the hematologists. The study was performed at the first affiliated hospital of Guangxi Medical University. Restrospective review of medical records, coded exchange of clinical data, SF levels and MRI examininations were authorized by the local institute. All of patients provided their written informed consent to participate in this study. Adults (≥18) finished the informed consent by themselves. And for minors (<18), the informed consents were obtained from the guardians on the behalf of the minors participants involved in this study. The study was approved by the Medical Ethics Committee of First Affiliated Hospital of Guangxi Medical University (the approval number: NO. 2008(KY-002).

### Serum Ferritin

Laboratory blood tests were performed 2 weeks after the last blood transfusion, followed by MRI evaluations with a 1-month interval in all patients. Measurements were carried out by an Electrochemiluminescence immunoassay (COBASE E 601, Roch, USA).

### MRI Protocols

Patients were scanned with a 1.5T MRI scanner (Siemens Avanto, Siemens Medical Systems, Erlangen, Germany) with the combination of body-matrix and spine-matrix surface coils. To ensure quality of the work, the local staff involved in this study (technicians, radiologists, and hematologists) received intensive onsite training from CMR specialists of Royal Brompton Hospital (London, UK). Each scan included the measurement of heart T2* and left ventricular ejection fraction (LVEF) using previously published techniques [14], [15], [16]. For T2* imaging, a single short axis mid-ventricular slice was acquired at eight echo times (2.97–21.68 ms) using a gradient-echo sequence within a single breath-hold. For myocardial T2* measurement, a homogeneous full thickness region of interest was chosen in the septum and signal intensities at lengthening echo times analysed using a truncation model [17]. LVEF were determined from steady-state free precession cines using CMR tools (Cardiovascular Imaging Solutions, London, UK).

### Statistical Analysis

Data were expressed as mean± one standard deviation, or median (range) as appropriate. Mann Whitney U test or t test were used for comparison of continuous variables between groups. The significance of the correlation between parameters was assessed using Spearman rank as the data was not normally distributed. Categorical data were compared using the chi-square test. Multivariate analysis used multiple linear regression analyses. Statistical analyses were carried out using SPSS Statistics 13.0 (SPSS Inc., USA). A p value less than 0.05 was considered statistically significant, and all p values were two sided.

## Results

### Patients

The main characteristics of these patients along with the main results of the study were summarized in Table 1.

**Table 1:** The main characteristics of Chinese TM patients along with the main results of the study.

	Patients(n = 201)
Male [n (%)]	125(62.2)
Ethnicity	
Han	161
Other	40
Median age (range),years	9(4–25)
Age group, years	
<6 [n (%)]	14(7)
6–<10 [n (%)]	89(44.2)
10–<15 [n (%)]	79(39.3)
15–<20 [n (%)]	14(7)
20–<25 [n (%)]	5(2.5)
Previous chelation therapy, [n (%)]	
DFO	42(20.9)
DFP	25(12.4)
DFO+DFP^a^	95(47.3)
DFX	8(4)
DFO+DFX^b^	20(9.9)
None	11(5.5)
Mean ± SD number of transfusions	116.3±91.4
Median SF ng/mL (range)	4536(524.7–23,640)
SF category [n (%)]	
<2500 ng/mL	36(17.9)
≥2500 ng/mL	165(82.1)
cardiac T2*, ms	25.5±12.5
T2* category [n (%)]	
<10 ms	26(12.9)
10–20 ms	42(20.9)
>20 ms	133(66.2)
LVEF, [%]	64.8±5.6
LVEF category [n]	175
<56% [n (%)]	6(3.4)
≥56% [n (%)]	169(96.6)

**DFO**,deferoxamine; **DFP**, deferiprone; **DFX**, deferasirox; **SD**, standard deviation; **SF**, serum ferritin; **LVEF**, left ventricular ejection fraction.

**^a^:** patients received both DFO and DFP as prior chelation therapies, but these may not have been in combination.

**^b^:** patients received both DFO and deferasirox as prior chelation therapies, but these may not have been in combination.

### Serum Ferritin

The median SF level of the patients was 4536 (525–23,640) ng/ml. The SF was >2500 ng/ml in 165 patients (82.1%). There was a significant correlation between SF and the total transfusion units (r = 0.175, p = 0.013). A similar result was found in the correlation between SF and age (r = 0.144, p = 0.042).

### Cardiac T2*

**Figure 1:**
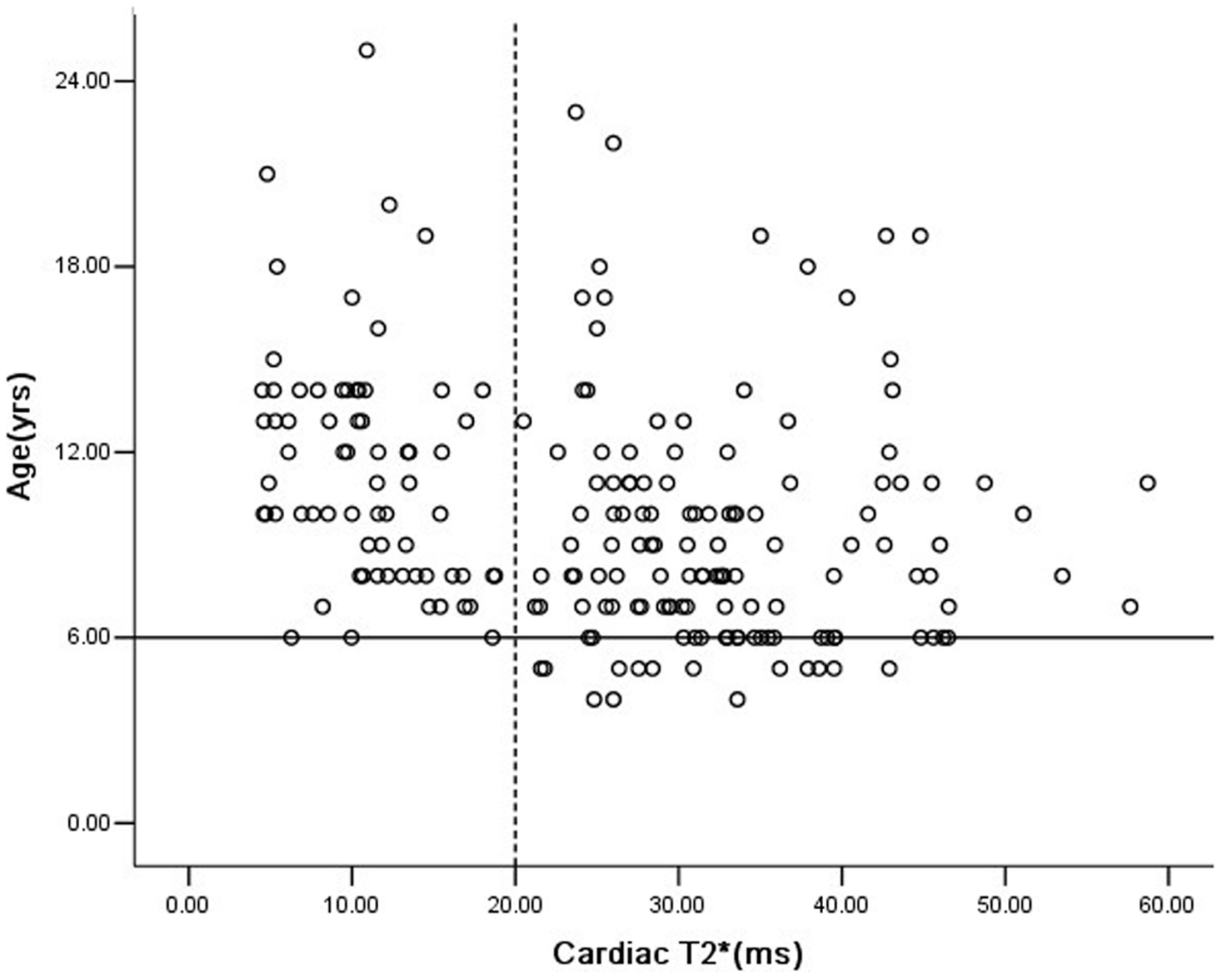
Correlation between cardiac T2* and age in Chinese TM patients. The dotted line represents cardiac T2* of 20 ms. The solid line represents age of 6 years old. Cardiac T2* was negatively associated with age (r = −0.328, p = 0.000) in these patients.

**Figure 2:**
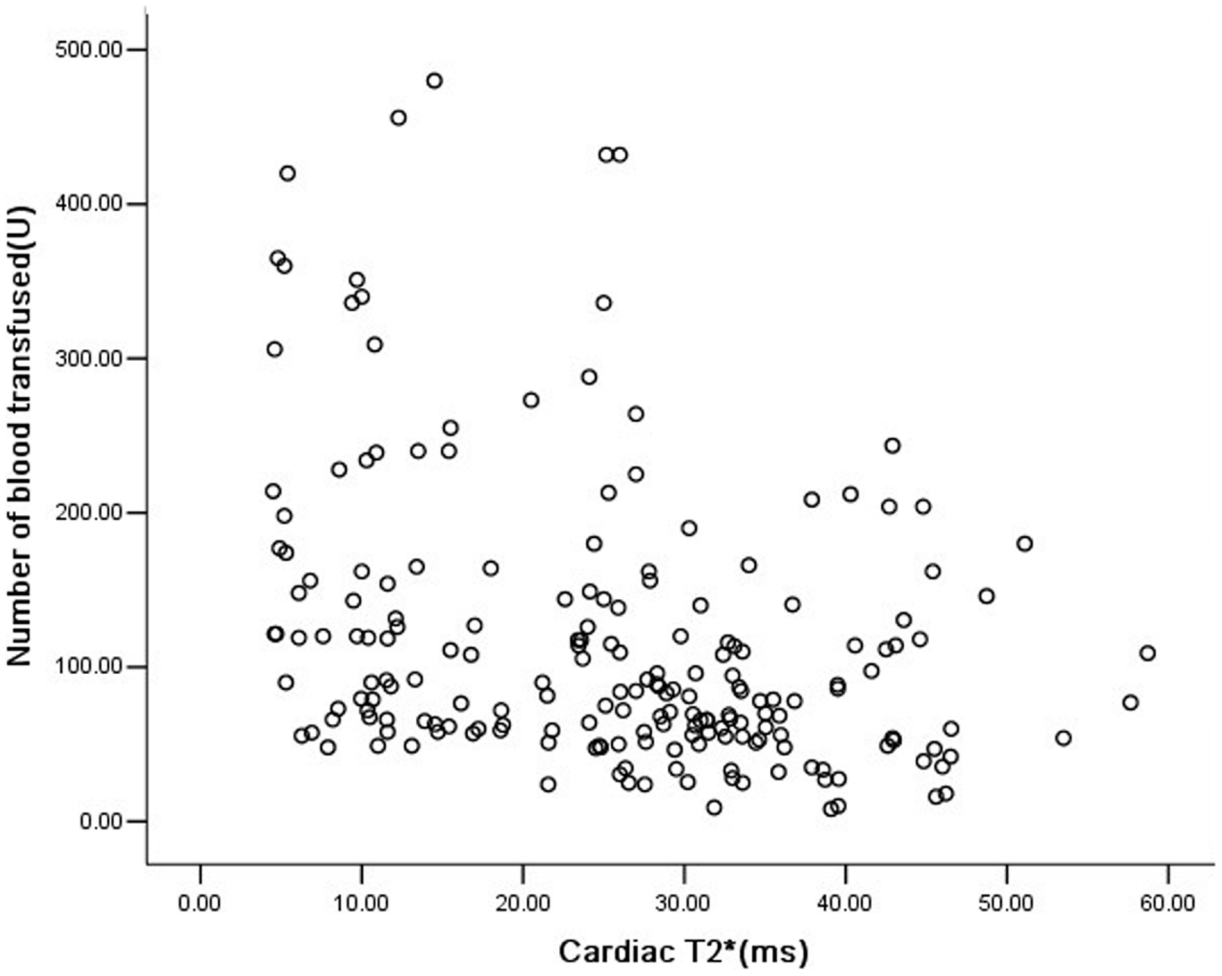
Correlation between cardiac T2* and total units of blood transfused in Chinese TM patients. Cardiac T2* was negatively associated with units of transfused blood(r = −0.360, p = 0.000).

**Figure 3:**
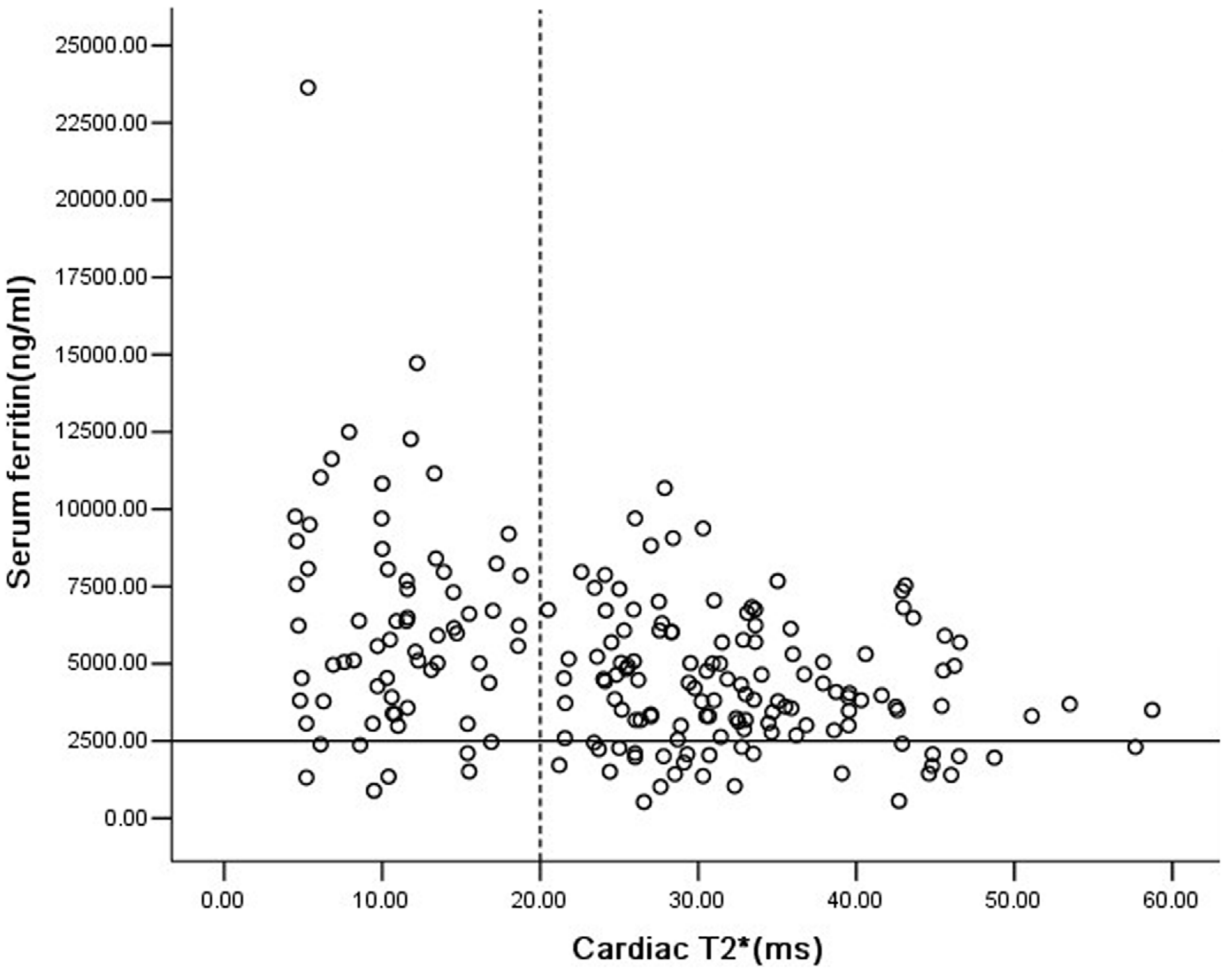
Correlation between cardiac T2* and serum ferritin(SF) values in Chinese TM patients. Cardiac T2* was negatively associated with SF (r = −0.319, p = 0.000). The dotted line represents reference range for cardiac T2* of 20 ms, and the solid line represents SF of 2500 ng/ml.

The mean cardiac T2* value was 25.5±12.5 ms (4.5–58.7 ms) for all 201 patients. Cardiac T2*≤20 ms were detected in 68 patients (33.8%), and cardiac T2*<10 ms were detected in 26 patients (12.6%). For patients ≤10 years old (n = 113), 32 patients (28.3%) had cardiac T2*≤20 ms, 3 of whom were only 6 years old. Fourteen patients of 5 years of age and younger were scanned, all with normal cardiac T2*>20 ms (Table 2). The five well-chelated patients all had cardiac T2*>20 ms. The mean cardiac T2* and incidence of cardiac T2*<10 ms in 161 Han ethnicity patients was not significantly different with 40 other ethnicity patients (25.8±12.7 vs 23.6±11.9, p = 0.311;33.5% vs 37.5%,p = 0.637).Cardiac T2* was negatively associated with age (Fig. 1, r = −0.328, p = 0.000) and the total units transfused (Fig. 2, r = −0.360, p = 0.000), respectively. A similar result was found in the correlation between cardiac T2* and SF (Fig. 3, r = −0.319, p = 0.000). Multivariate analysis confirmed the negative correlation between cardiac T2* and total units transfused (β = −0.297, P = 0.006), and SF (β = −0.234, P = 0.000), but the non-correlation between cardiac T2* and age (β = −0.081, P = 0.421).

**Table 2:** T2* information of patients divided into five-year age groups.

T2* category	Age groups, n (%)				
	≤5 y	6–10 y	11–15 y	16–20 y	>20 y
>20 ms	14(100)	81(71.7)	27(48.2)	9(64.3)	2(50)
10–20 ms	0	23(20.3)	14(25.0)	4(28.6)	1(25)
<10 ms	0	9(8.0)	15(26.8)	1(7.1)	1(25)

### Left Ventricle Ejection Fraction (LVEF)

**Figure 4:**
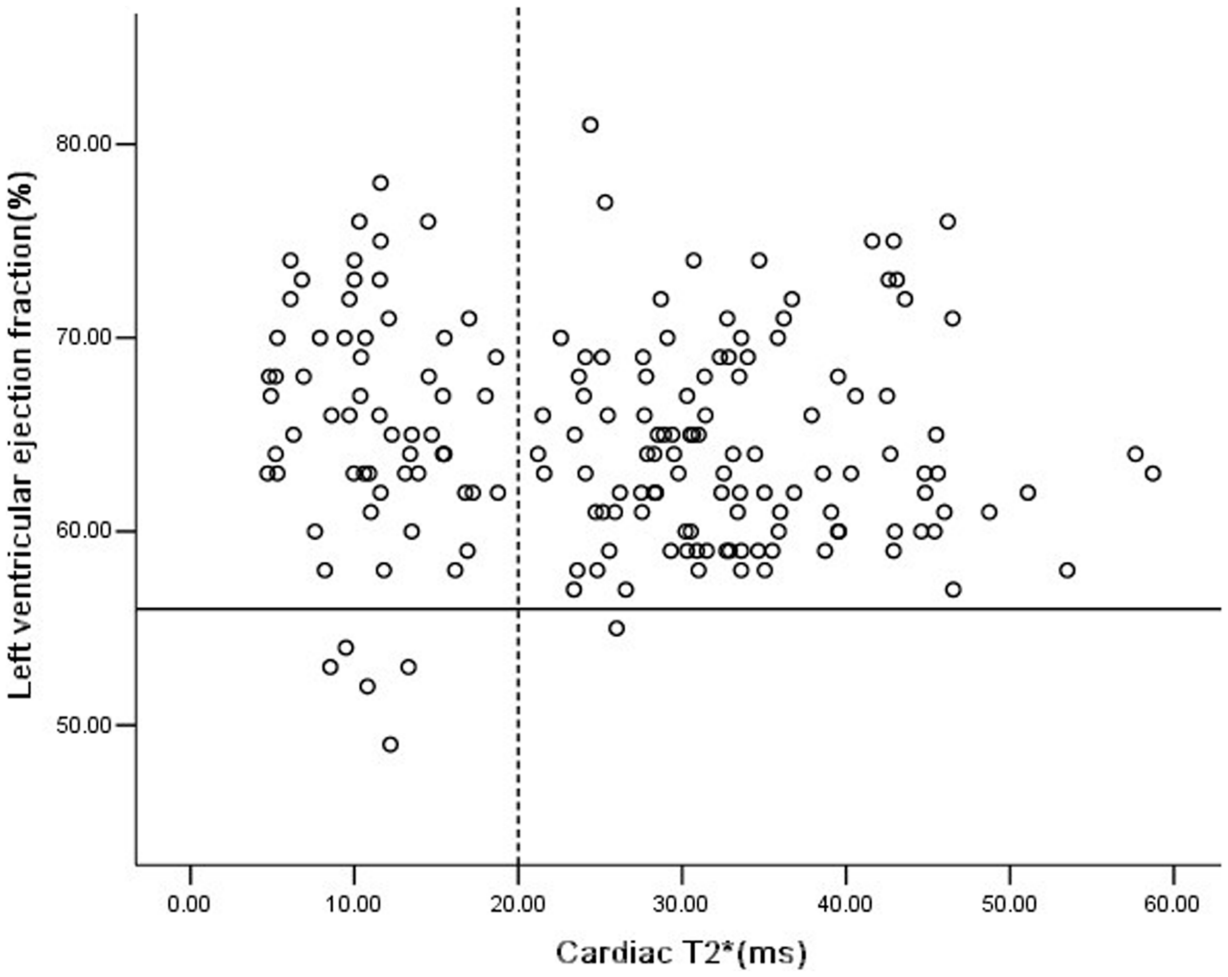
Correlation between cardiac T2* and left ventricular ejection fraction (LVEF) in Chinese TM patients. No correlation was found between cardiac T2* and LVEF (r = −0.147, p = 0.052). The solid line presents reference range (56%) for LVEF. The dotted line represents reference range for cardiac T2* of 20 ms.

LVEF was measured by CMR in 175 patients and the median of LVEF was 64% (49%–81%). Twenty-six young patients failed to measure LVEF because they could not comply sufficiently with breath-hold instructions. Six patients (3.4%) had LVEF<56% [6]. The median LVEF of 62 patients with cardiac iron overload (T2*<20 ms) was not significantly different from those 113 patients without cardiac iron overload (T2*>20 ms) (65.5% vs 63%, p = 0.146). LVEF<56% was seen in 5 patients (8.1%) with cardiac iron overload, comparing with only 1 (0.9%) without cardiac iron overload (p = 0.021. Three patients with LVEF<56% were under 10 years old. No correlation was found between LVEF and SF (r = 0.111, p = 0.145) or LVEF and cardiac T2* (r = −0.147, p = 0.052) in these 175 patients (Fig. 4).

### Cardiac Disease

A patient requiring cardiac medications, or with a LVEF<56%, or with persistent arrhythmia [18], [6] was considered to have a cardiac disease. Ten patients (5%) had cardiac disease, 3 with heart failure hospitalization, 1 with junctional rhythm, and 6 with impaired LV systolic function (LVEF<56%). Among them, 8 patients had cardiac iron overload; of the two patients with normal cardiac iron, one had myocarditis and the other junctional rhythm disturbance. None of the well-chelated patients had cardiac involvement. Four patients with cardiac involvement were under 10 years old. Three heart failure patients had cardiac iron overload but LVEF>56% (63%, 68% and 68%). The patient with junctional rhythm had both a normal cardiac T2* and a normal LVEF.

## Discussion

Iron induced cardiomyopathy can be reversed if aggressive chelation begins early [19], [20]. Thus, early detection of myocardial iron deposition is imperative to prevent overt heart failure. In non-chelated patients receiving regular transfusions, cardiomegaly develops by the age of 10 years and heart failure by the age of 16 years [21]. The duration of life after the onset of failure was less than 3 months in over half of the patients. Clinical diagnosis is often delayed due to the typically late onset of symptoms. Iron overload in TM occurs due to a combination of repeated blood transfusions and excessive gastrointestinal absorption. Little is known about the natural history of iron deposition in the heart. Previous studies suggested that myocardial deposition takes place after a minimum of 75 blood transfusions [22], [23]. Assessment of myocardial iron is essential clinically but conventional noninvasive techniques are less than ideal. Endomyocardial biopsy is invasive and not reliable because myocardial iron deposition is not homogeneous [24], [25]. Even the combination of SF, liver iron concentration and ventricular function can only detect heart failure at a late stage [26], [27], [28]. Usefully, cardiac T2* can be used to monitor cardiac iron overload non-invasively, reproducibly and accurately. However, cardiac T2* data on children younger than 10 years is rather limited. Wood et al recently reported from a cohort of 77 TM patients in Italy and US that no cardiac iron was observed under the age of 9.5 years [29]. In another earlier report, it was suggested that cardiac iron overload occurred only after at least 13 years of chronic transfusion therapy [18]. These studies however, were based on well-chelated patients. Fernandes et al recently reported a single patient with cardiac iron overload at the age of 7 years but this study was limited to only 23 patients with TM and other forms of anemia [30]. In our study, the vast majority of patients (97.5%) had not received a good standard of chelation therapy. Cardiac iron overload was found in patients under 10 years old with severe cardiac iron overload in a proportion, associated with impaired LVEF and cardiac symptoms. We detected severe myocardial siderosis in patients as young as 6 years old.

The median age of our TM patients was 9 (4–25) years old. The median SF level was 4536 ng/ml and 165 patients (82.1%) had SF>2500 ng/ml. The data confirmed our previous finding that Chinese TM patients were younger and had greater burden of iron overload than those in western countries [31]. Myocardial iron overload was detected in 33.8% of our patients. This value was lower than that reported in west countries, where cardiac T2*<20 ms were seen in 32%–65% of adults with TM [32], [33], [16]. One explanation was that our patients were much younger and most of them were children, but it is also notable that the average yearly units transfused was only 13 per patient. SF was positive associated with age. Cardiac T2* was negatively associated with age and with SF. This finding was in contrast to other reports showing no relation between cardiac T2* and SF in patients chelated from a young age with a variety of different regimes, in which the varying regimes may have resulted in the loss of relationship between cardiac T2* and SF. For instance it is recognized that DFO results in preferential chelation from the liver whereas Deferiprone may be more effective for chelation of cardiac iron. The younger age and lower levels of chelation therapy may account for the discrepancy between our and previous studies [34], [35], [33].

To our knowledge, this was the first cardiac T2* study on a large and poorly-chelated and modestly transfused TM population. The results may help develop guidelines to the clinical assessment and management of iron overload in TM patients in developing countries. It is estimated that more than 300,000 children are born annually with severe inherited disorders of hemoglobin and approximately 80% of these births occur in low- or middle-income countries [36], [37]. Over the next 20 years approximately 100,000 cases of Hb E/βthalassaemia will be added to the Thailand population and 20,000 beta TM babies will be born each year in China [38], [39]. It is a global public health challenge particularly in developing countries. In low- or middle-income countries, many TM children receive inadequate iron chelating therapy and many of them die as teenagers due to the effects of iron overload [10]. Bejaoui M et al studied 391 TM patients in Tunisia. They found that the mean age of death was 10.48 years with heart failure as the major cause of death [40]. A disturbing study of 106 TM patients in Baise City (Guangxi,China), showed that 85 patients (80%) died before the age of 5 years [11]. At present, TM patients older than 30 years old are seldom found in mainland China. The current life expectancy of TM patients is unclear. While progress has been made for the control and genetic screening for thalassemia trait over the last decade in China, health care for patients suffering with TM in China continues to pose a great challenge to the public health services. Many factors, including the lack of disease specific knowledge, shortage of blood, limited availability of chelators, lack of specialist physicians and the limited resources within the health insurance system pose challenges to optimum care. Although health insurance coverage has increased dramatically over the last decade in China, from 15% in 2000 to 96% in 2011, the financial protection remains insufficient [41]. Although DFO has been used for over 40 years in Western countries, it has only been available in China since 1998. DEP and DFX were introduced to China in 2003 and 2010, respectively. Further, CMR T2* was first introduced to China in 2010 and only a small number of TM patients have benefitted from this technique. In brief, developing countries lag far behind developed countries in the control and management of thalassemia. It is therefore vital that international health agencies and governments of countries where these diseases are common start to develop the partnerships between rich and low-income countries and establish specific guidelines towards the most economic and effective approach to control and manage thalassemia.

In conclusion, we present the findings of a large cohort of Chinese TM children and adolescents. The vast majority of patients are poorly-chelated and as a result of relative chelation naivety, clear associations can still be demonstrated between cardiac iron stores and age and serum ferritin. Myocardial siderosis is strongly associated with impaired LVEF and heart failure. Our results show the effects of poor chelation of young patients and that the first assessment of cardiac T2* should take place as early as 6 years of age.
